# Assistive Technologies to Guide the Management of Volume Overload in Hemodialysis Patients: Are They Ready for Implementation in Daily Clinical Practice?

**DOI:** 10.3390/life16030380

**Published:** 2026-02-27

**Authors:** Panagiotis I. Georgianos, Vasileios Kamperidis, Christodoula Kourtidou, Athanasios Roumeliotis, Konstantinos Leivaditis, Vassilios Liakopoulos

**Affiliations:** 1 2nd Department of Nephrology, AHEPA Hospital, Aristotle University of Thessaloniki, 54636 Thessaloniki, Greece; 2 1st Department of Cardiology, AHEPA Hospital, Aristotle University of Thessaloniki, 54636 Thessaloniki, Greece

**Keywords:** volume overload, bioimpedance spectroscopy, lung ultrasound, relative blood volume monitoring, hemodialysis

## Abstract

Hypervolemia in hemodialysis patients is often covert and physical examination is frequently inaccurate in diagnosing the presence of subclinical volume expansion. Newer technologies, such as relative blood volume monitoring during dialysis, bioimpedance spectroscopy and lung ultrasound, offer promise for a more accurate assessment of volume status in these patients. Prospective observational studies support the notion that hypervolemia, as assessed with these assistive methods, is a strong and independent predictor of adverse cardiovascular outcomes and mortality in hemodialysis patients. However, randomized controlled trials and meta-analyses have failed to demonstrate that as compared with usual care, a volume management strategy based on the use of these assistive techniques is more effective in mitigating the risk of adverse intradialytic events and in improving long-term cardiovascular outcomes. In this article, we explore the question: whether assistive technologies are ready for implementation in daily clinical practice for the long-term management of volume overload in hemodialysis patients. We critically evaluate evidence from recently completed randomized trials and provide directions for future research in this important scientific area.

## 1. Physical Examination for the Evaluation of Volume Status in Hemodialysis Patients

Hypervolemia in hemodialysis patients is very common and can be easily recognized when these patients present emergently with overt signs and symptoms of acute decompensated heart failure [1]. However, the most common scenario in daily clinical practice is that overhydration remains covert, aggravating in this way the long-term risk for uncontrolled hypertension, worsening of heart failure and adverse cardiovascular outcomes [1].

Several sources of evidence suggest that a careful physical examination is unreliable to discriminate hypervolemia from euvolemia [2,3,4]. As an example, in a cross-sectional analysis of 146 asymptomatic hemodialysis patients, the presence of pitting pedal edema was associated with a variety of cardiovascular risk factors (i.e., age, body mass index, left ventricular hypertrophy) [2]. However, in multivariate-adjusted models, several “objective” measures of volume excess, such as inferior vena cava diameter in expiration, relative blood volume (RBV) monitoring, plasma volume markers and inflammation markers were not independent determinants of pedal edema [2]. In the DRIP (Dry-weight Reduction in Hypertensive Hemodialysis Patients) trial [5], 150 hypertensive hemodialysis patients without clinically overt hypervolemia were randomly assigned in a 2:1 ratio to an ultrafiltration group and a control group. Over a follow-up period of 8 weeks, as compared with no intervention in the control group, probing of dry-weight in the ultrafiltration group provoked a reduction of −6.6/−3.3 mmHg in 44-h interdialytic ambulatory blood pressure (BP) [5]. The BP-lowering effect of dry-weight reduction was similar in magnitude in patients with and in patients without pitting pedal edema in physical examination [5]. Therefore, the presence of pedal edema, a clinical sign of volume excess, failed to predict the responsiveness of hypertensive hemodialysis patients to an intervention targeting the achievement of an adequately low postdialysis weight.

If the discriminatory power of physical examination is suboptimal, there is therefore a critical unmet need for the development of more reliable methods for the assessment and long-term management of volume status in hemodialysis patients. Several techniques, such as intradialytic RBV monitoring, bioimpedance spectroscopy and lung ultrasound, are currently under investigation, offering promise that their implementation in daily clinical practice may assist the nephrologist in optimizing volume control and in improving long-term clinical outcomes in this high-risk patient population [6]. These techniques may be superior to physical examination in identifying hemodialysis patients with subclinical volume excess. Accordingly, there has long been a hypothesis that assistive technologies can possibly guide therapy in such high-risk patients. In the following sections of this article, we explore the question whether the broad clinical utilization of these assistive technologies is currently supported by a solid body of evidence.

## 2. Observational Studies

Large observational studies have provided data that hypervolemia, as assessed more “objectively” with the use of assistive technologies, is a strong and independent predictor of all-cause death risk in patients on hemodialysis [7,8,9]. Sustained exposure to volume excess over a long-term period possibly further magnifies the risk for adverse clinical outcomes. This was illustrated in a cohort study that included 39,566 patients with incident kidney failure [10]. Baseline and cumulative exposure to hypervolemia over 1 year were assessed using the technique of whole-body bioimpedance spectroscopy. Baseline overhydration was independently associated with all-cause mortality, an association that was consistent across all systolic BP categories [hazard ratio (HR): 1.51, 95% confidence interval (CI): 1.38–1.65 for patients with systolic BP < 130 mmHg; HR: 1.25, 95% CI: 1.16–1.36 for patients with systolic BP 130–160 mmHg; HR: 1.30, 95% CI: 1.19–1.42 for patients with systolic BP > 160 mmHg] [10]. Sustained 1-year-long exposure to hypervolemia was associated with an even higher all-cause death risk. Once again, this risk association was persistent irrespective of the severity of hypertension (HR: 1.94, 95% CI: 1.68–2.23 for those with systolic BP < 130 mmHg; HR: 1.51, 95% CI: 1.35–1.69 for those with systolic BP 130–160 mmHg; HR: 1.62, 95% CI: 1.39–1.90 for those with systolic BP > 160 mmHg) [10]. However, these data should be interpreted with caution, taking into consideration that studies following an observational design cannot establish direct cause-and-effect risk associations.

## 3. Clinical-Trial Evidence

Individual randomized controlled trials comparing a volume management strategy guided by the use of assistive technologies with usual care have been few in number and have provided contradictory results [11,12]. In a 2017 meta-analysis of seven trials (involving 1312 dialysis patients), as compared with usual care, a bioimpedance spectroscopy-based strategy was associated with a small, but statistically significant reduction in overhydration index [weighted mean difference (WMD): −0.43 L; 95% CI: −0.71 to −0.15] [13]. However, this improvement in volume status was not translated into a long-term benefit of this strategy on all-cause mortality [relative risk (RR): 0.87; 95% CI: 0.54–1.39] [13]. In a larger 2019 meta-analysis of 12 trials (incorporating data from a total of 2406 dialysis patients), the use of assistive technologies was not superior to usual care in lowering the all-cause death risk (RR: 0.92; 95% CI: 0.57–1.51) [14]. Furthermore, a volume management strategy based on the guidance of assistive technologies had little to no benefit on a number of surrogate endpoints, such as BP control, regression of left ventricular hypertrophy, improvement in symptoms or in the incidence of intradialytic hypotension [14]. The strength of evidence provided by these two meta-analyses was low-to-moderate [13,14]. The vast majority of the included studies had a high or an uncertain risk of bias due to inherent methodological limitations, such as small sample sizes, short duration of intervention, and evaluation of surrogate endpoints instead of “hard” clinical outcomes as primary efficacy measures.

In the more recent LUST (Lung Water by Ultrasound-Guided Treatment in Hemodialysis Patients) trial [15], 367 hemodialysis patients at high risk for adverse cardiovascular events were randomized in a 1:1 ratio to an active arm and a control arm (Table 1). In the active arm, the lung ultrasound B-line score guided the intensification of ultrafiltration during dialysis and up-titration of drug treatment. In the control arm, patients received standard-of-care treatment. Over a mean follow-up of 1.49 years, lung congestion was more frequently relieved in the active arm (78%) than in the control arm (56%) [15]. However, a lung ultrasound-guided strategy for the management of volume status failed to improve the prespecified primary outcome, defined as the composite of all-cause death, non-fatal myocardial infarction or worsening of heart failure (HR: 0.88; 95% CI: 0.63–1.24) [15]. The risks of all-cause hospitalization (HR: 1.03; 95% CI: 0.77–1.36) and cardiovascular-related hospitalization (HR: 1.02; 95% CI: 0.71–1.46) did not differ between the two arms [15]. Furthermore, a lung ultrasound-based strategy was not associated with a significant improvement in patient-reported symptoms or health-related quality of life [15]. Once again, the lower than originally planned recruitment rate and the short duration of follow-up possibly limited the opportunity to detect a statistically significant benefit of this intervention on the primary and secondary outcomes of the LUST trial. However, it has to be mentioned that a benefit of a lung ultrasound-guided therapy may truly exist. A larger and longer-term clinical trial with a careful design is needed to confirm or refute the superiority of this strategy over standard-of-care in the future.

## 4. Conclusions and Directions for Future Research

In conclusion, assistive techniques, such as RBV monitoring, bioimpedance spectroscopy and lung ultrasound are not time-consuming, do not require long-term training of the care giver and can be easily performed at the bedside of the patient. Several longitudinal studies have shown that volume excess in patients receiving maintenance dialysis, as assessed with the use of these technologies, is strongly associated with a heightened risk for adverse outcomes [7,8,9,10]. However, the currently available clinical trials have failed to prove the superiority of a volume management strategy guided by the use of assistive technologies relative to usual care in improving “hard” clinical outcomes and several other intermediate endpoints [13,14,15]. A carefully designed clinical trial is urgently warranted to fully elucidate this crucial issue in the upcoming future. The first step is as follows: in a trial including clinically euvolemic hemodialysis patients with poorly controlled hypertension, those randomly assigned to the control arm would have their dry-weight probed gently and gradually with the guidance of intradialytic symptoms. In the intervention arm, probing of dry-weight would be based on the guidance of an assistive technique. The primary research hypothesis is that the incidence of intradialytic symptoms and adverse events would be lower in the intervention arm than in the control arm. We do not expect a significant between-arm difference in other outcomes, such as BP control or regression of left ventricular mass index. We expect a greater relief of volume overload with fewer adverse events and improvement in health-related quality of life. The second step is the design of a fully dedicated, multi-center, randomized controlled trial adequately powered to assess significant between-arm differences in the incidence of adverse cardiovascular events and all-cause mortality. In anticipation of more definitive results from future randomized controlled trials in this area, the broad utilization of assistive technologies as a tool to guide therapy in hemodialysis patients is not evidence-based. We continue to follow the standard-of-care approach in the long-term management of volume status in our patients on hemodialysis. Accordingly, in those with uncontrolled interdialytic hypertension, we probe dry-weight with a careful monitoring of symptoms and responsiveness of patients to treatment.

## Figures and Tables

**Table 1:** Design and main results of the LUST trial.

Parameter	LUST Trial [15]
Design	International, multi-center, open-label RCT
Patient characteristics	Patients receiving chronic hemodialysis with high CV risk
N	367
Intervention	Predialysis lung ultrasound-guided titration of ultrafiltration during dialysis and drug treatment vs. usual care
Follow-up	1.59 years (mean)
Key findings	▪ More frequent relief of lung congestion in the active arm than in the control arm (78% vs. 56%, respectively). ▪ No between-arm difference in the primary outcome, defined as the composite of CV death, non-fatal MI or decompensated HF (HR: 0.88; 95% CI: 0.63–1.24). ▪ No between-arm difference in the incidence of all-cause hospitalization (HR: 1.03; 95% CI: 0.77–1.36) and CV-related hospitalization (HR: 1.02; 95% CI: 0.71–1.46).
Limitations	▪ Lower than originally planned recruitment rate. ▪ Short duration of follow-up.
Interpretation	As compared with usual care, a lung ultrasound-guided strategy resulted in better relief of lung congestion. However, this strategy was not translated into a long-term benefit on “hard” CV outcomes.

Abbreviations: CI = confidence interval; CV = cardiovascular; HF = heart failure; HR = hazard ratio; LUST = Lung Water by Ultrasound-Guided Treatment in Hemodialysis Patients; MI = myocardial infarction.

## Data Availability

No datasets were analyzed or generated for the preparation of this manuscript.

## Abbreviations

The following abbreviations are used in this manuscript:

DRIP	Dry-weight Reduction in Hypertensive Hemodialysis Patients
BP	Blood Pressure
RBV	Relative Blood Volume
CI	Confidence Interval
HR	Hazard Ratio
RR	Relative Risk
WMD	Weighted Mean Difference
LUST	Lung Water by Ultrasound-Guided Treatment in Hemodialysis Patients
HF	Heart Failure
CV	Cardiovascular
MI	Myocardial Infarction
